# THz Sensing of Human Skin: A Review of Skin Modeling Approaches

**DOI:** 10.3390/s21113624

**Published:** 2021-05-23

**Authors:** Jiarui Wang, Hannah Lindley-Hatcher, Xuequan Chen, Emma Pickwell-MacPherson

**Affiliations:** 1 Department of Electronic Engineering, The Chinese University of Hong Kong, Shatin, Hong Kong 999077, China; wangjr3399@link.cuhk.edu.hk (J.W.); xuequanchen@cuhk.edu.hk (X.C.); 2 Department of Physics, University of Warwick, Coventry CV4 7AL, UK; Hannah.Hatcher@warwick.ac.uk

**Keywords:** Terahertz spectroscopy, in vivo, skin, skin modeling

## Abstract

The non-ionizing and non-invasive nature of THz radiation, combined with its high sensitivity to water, has made THz imaging and spectroscopy highly attractive for in vivo biomedical applications for many years. Among them, the skin is primarily investigated due to the short penetration depth of THz waves caused by the high attenuation by water in biological samples. However, a complete model of skin describing the THz–skin interaction is still needed. This is also fundamental to reveal the optical properties of the skin from the measured THz spectrum. It is crucial that the correct model is used, not just to ensure compatibility between different works, but more importantly to ensure the reliability of the data and conclusions. Therefore, in this review, we summarize the models applied to skin used in the THz regime, and we compare their adaptability, accuracy, and limitations. We show that most of the models attempt to extract the hydration profile inside the skin while there is also the anisotropic model that displays skin structural changes in the stratum corneum.

## 1. Introduction

### 1.1. Terahertz Radiation and Systems

Terahertz (THz) waves lie between 0.1 and 10 THz (1THz = 10^12^ Hz), corresponding to wavelengths ranging from 30 μm to 3 mm. The rapid development of THz technology in the last three decades has promoted numerous applications in communication, security, biosensing, aerospace etc. Among them, biomedicine has long been considered a promising application area [1]. One important reason is the non-ionizing and non-invasive nature of THz radiation, making it a safe modality for biomedical in vivo imaging. Another key factor comes from the high absorption of water, which despite limiting the depth of penetration, provides high sensitivity to the water content in living tissues. Given these characteristics, THz in vivo studies have been mainly focused on skin, as THz waves can penetrate through the superficial layer, and the measured THz response is sensitive to its water concentration and tissue structure. 

The aim of skin measurements is to investigate the morphology, histology, functions and properties [2]. Moreover, it is of great interest to apply skin imaging and measurements for the diagnosis of skin lesions and pathological processes objectively and quantitively [2,3]. A wide range of imaging methods are currently used. Histological examination of biopsies usually combined with optical microscopy is invasive and requires sample preparation and fixation which can change the biological properties of the sample but is the gold standard to reveal pathological changes in tissues [4]. Electron microscopy provides especially high resolution at nanometer level. However, sample preparation is required to enhance the contrast [5]. To meet the need for non-invasive in vivo measurements of skin, emerging techniques are now available or under research. Fluorescence microscopy is another kind of optical microscope based on fluorescence and phosphorescence and combined with confocal laser scanning microscopy, it has been widely used for evaluating transdermal drug delivery [6]. It is able to track and quantitatively analyze drugs labelled with fluorescent dyes. Near infrared(NIR) imaging is another commonly used method for skin measurement, it can also be used for hydration sensing because of the clear absorption of water molecules at 1450 and 1920 nm [7]. However, NIR imaging usually yields complex spectra which are difficult to interpret. Raman spectroscopy combined with confocal microscopy can provide information about the chemical components and concentration distribution through the depth of the skin, but it is limited by the slow imaging speed, low sensitivity, and sophisticated data analysis required [8,9]. Optical coherence tomography (OCT) is another method that can be used to measure the skin, but it primarily reveals morphological changes in the skin [10,11]. Computed tomography (CT) and magnetic resonance imaging (MRI) are other commercialized medical imaging techniques, however, they both have limitations. CT involves X-ray radiation which is ionizing [12,13] and MRI is best suited to imaging internal soft tissue though there is research investigating the application of this technique to skin imaging, but it is still at a preliminary stage [14]. Based on dielectric differences of normal and malignant tissues, microwave and millimeter-wave technologies also provide cost-effective options for tumor diagnosis [15,16]. It has been reported that millimeter waves are also sensitive to the water and thickness variation of skin and could be a potential technique for skin diagnosis [17,18]. Compared to THz waves, millimeter waves have a deeper penetration depth in living tissues of over 1 mm, reaching down to the dermis layer [17,18]. However, the longer wavelengths also restrict the spatial resolution limit for standard imaging configurations. Based on the sensitivity of THz radiation to water and its penetration depth (100 μm to several mm) into skin and tissues, THz sensing could provide superficial information and is therefore suitable for skin measurements. THz sensing probes the intermolecular vibrations of water and other biomolecules, while NIR measurements are dominated by intramolecular vibrations. Given the high sensitivity of THz light to water content, normal tissues and cancerous tissues can be differentiated. Moreover, the picosecond-level time-resolved ability of THz pulsed imaging enables a depth resolution of ~100 μm, comparable to 50–100 μm level of MRI [19]. Combined with appropriate skin modelling which will be detailed in Section 2, better depth resolutions down to few tens of micrometers could be achieved. Compared to OCT which mainly reveals the structure and morphology of tissues, THz imaging is sensitive to both the structural and chemical properties. Therefore, THz imaging is a promising technique for quantitative in vivo skin analysis, which could aid the diagnosis of skin lesions and pathological processes. However, various technical challenges need be overcome before THz techniques can be robustly adopted in a clinical setting. The current THz systems still suffer from low imaging speeds, limited measurement flexibility and critical optical alignment. The further development of THz devices and systems can gradually pave the way to its utility and acceptance in wider applications. 

**Figure 1:**
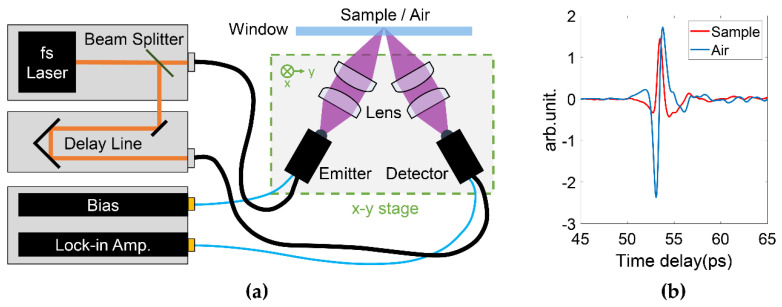
(**a**) Typical THz pulsed laser imaging system in reflection geometry. The THz optical system is assembled on a x-y 2D stage to enable raster scanning the sample. (**b**) Examples of the THz pulses reflected from the quartz-volar forearm and quartz-air interfaces, respectively.

For THz in vivo skin measurements of humans, reflection geometry is required as tissues highly attenuate the THz radiation [20]. Various THz devices can be adopted to perform reflection measurements. For example, laser feedback interference in quantum cascade lasers is a promising technique for biomedical imaging [21]. Such a mechanism not only provides a high resolution due to the short wavelength (frequency typically > 2 THz), but also a good signal-to-noise ratio originating from the coherent nature of the interference. Rakić et al. have successfully employed this technique to image porcine tissues and murine skin [22,23]. Similar setups can be adapted to in vivo measurements. THz time-domain spectroscopy (TDS) is the most widely used technique for in vivo studies. Figure 1a shows a typical THz reflection-mode TDS system based on fiber-coupled photoconductive antennas. In this system, the femtosecond pulse from the fiber laser is split and sent to the THz emitter and detector, respectively. On the emitter side, the input femtosecond laser pulse excites the free carriers on the semiconductor substrate. The carriers are accelerated by the biased voltage on the electrode and quickly recombine in a few picoseconds. The rapidly generated and annihilated carriers form a transient current, which then radiates an electromagnetic wave with its electric field proportional to the time-variation of the current. This radiation thus contains broadband THz frequencies given by its picosecond pulse width. The THz wave is then guided by the optics, reflected by the sample and collected by the detector. In the detector, the femtosecond pulse again excites the photocarriers, which are accelerated by the THz electric field to produce the photocurrent. The generated photocurrent in detector is then amplified. As the femtosecond pulse is over an order shorter than the THz pulse, the detected current is only proportional to the THz electric field at the moment it interacts with the THz wave. By moving the delay stage to change the optical path difference between the pumping and probing light, the whole THz waveform can be sampled in the time-domain. Figure 1b illustrated the examples of the THz time-domain waveforms reflected from the quartz-volar forearm and quartz-air interfaces, respectively. In such a setup, the THz image can be acquired by raster scanning the region of interest by either moving the window-sample system or the optical system. Figure 1a shows the latter approach. For imaging data, any model that applies for the skin characterization is then applied to the data at each pixel.

### 1.2. Biomedical Applications of THz Imaging

As previously introduced, in vivo THz studies have mainly focused on skin due to the shallow penetration depth. Investigations into utilizing THz imaging for diagnosis of cancer, scar measurements, monitoring drug diffusion and hydration sensing have been reported [24,25,26,27,28]. The origin of these applications is mostly based on the sensitivity to water. For example, THz imaging was shown to be capable of identifying cancerous regions as the higher water content and the structural changes of tumors compared to healthy tissue leads to an increased refractive index and absorption coefficient [29,30,31]. Wallace et al. used THz imaging to identify basal cell carcinoma (BCC) and the results showed high correlation with histology images [32]. Other investigations have also demonstrated that THz imaging is able to detect the boundary of breast and brain tumors [30,31]. 

Utilizing the excellent depth-resolving ability of THz-TDS, early work by Cole et al. showed that a single THz pulse is able to identify the stratum corneum (SC), the upper layer of human skin, and measure the change in thickness across different regions on the body [33]. This is also enabled by the water-content difference between the SC and the lower epidermis, as the SC is normally much less hydrated. However, for skin in other body sites, such as the volar forearm and wrist, this is not the case as the SC is so thin that the second reflection cannot be resolved. Scars are also found to have different water concentrations from healthy tissue. Fan et al. used THz imaging to monitor the human scar healing process and observed that the difference in the optical properties of scarred and healthy tissue are still distinguishable even after few months. This means that THz imaging could help monitor scar treatment and management [34]. Further work by Wang et al. used THz spectroscopy and imaging to evaluate the effect of treating human skin with silicone gel sheeting. This work indicated that THz imaging is able to detect subtle fluidic changes inside skin [35]. 

Drug diffusion is also accompanied with changes of the water concentration inside skin. Kim et al. used THz reflection imaging to monitor the transdermal drug delivery of ketoprofen and DMSO mixtures and show that THz imaging is able to differentiate different concentrations of drug solution and that the pulse information can reveal the depth of drug penetration [36,37]. Wang et al. show that THz imaging could be used as a label-free modality to evaluate the efficiency of different transdermal drug delivery methods including needle patches. They also revealed that the changes in the THz signal are caused by the drug solution displacing water inside skin, this means it is possible to extract the amount of drug solution that has penetrated into the skin [26]. 

### 1.3. Variables Affecting In Vivo THz Measurements of Skin 

In vivo measurements are a lot more complicated than ex vivo measurements, due to the complexity of living tissues, variations between different subjects, changes in the individual conditions etc. Variables that will affect the THz response should be carefully considered and well controlled during the measurement. Therefore, it is of vital importance to employ a robust experimental protocol to enable consistent in vivo measurements. For example, skin measurements are usually conducted in a reflection geometry with a window, either made of quartz or polymer, to help position the skin. Therefore, the contact pressure and occlusion by imaging window inevitably affect the result. Wang et al. found that the contact pressure between skin and the quartz window can significantly alter skin properties. A higher pressure applied to skin usually leads to a lower reflectance [38]. Another factor that needs to be considered is the occlusion effect. When skin is in contact with a window, water molecules can no longer evaporate to the outside of skin and accumulate in the SC, the water hydration inside the skin therefore increases. Sun et al. report how occlusion affects skin measurements and apply a biexponential model to describe the occlusion effect, making it possible to account for changes during raster scanning due to occlusion [39]. 

A comprehensive study about the variables that affect the THz response of skin and the protocol to control them has been presented by Lindley-Hatcher et al. [40] This protocol integrates pressure sensors with a THz-TDS system to provide real-time feedback on the pressures applied, and automatically starts the data acquisition when it falls within the specified pressure range. The pressure sensor output gives an indication of the start point for the measurement and this also enables a record of the occlusion time. A thorough protocol including a normalization method, consistent room temperature and humidity is proposed to account for natural variation of the skin between measurements and ensure that results from different subjects can be reliably compared. Consistent in vivo measurement results were achieved with the successful control of these variables.

### 1.4. Aim of this Review

The ability to use THz imaging and spectroscopy for different skin applications has been demonstrated through many studies. However, a unified model of skin for use in the THz range that unambiguously interprets the light–skin interactions has not yet been found, partially due to the complexity of living tissues and the divergent measurement protocols. Therefore, we present an overview of the applications of THz sensing of human skin with a focus on the model of skin used, and compare the adaptability, accuracy and limitations of these models. The models in this review consider the skin structure as a function of depth: the spatial variation in 2D imaging scan is not within the scope of this review.

## 2. New Advances of THz Measurement and Modeling of Skin

In this section, we focus on the optical models used in the THz regime for skin, including the dielectric model for describing the optical properties, and the structural models that describe the light-skin interaction. The former is related to the polarizing properties of different tissues of skin in response to the THz electromagnetic radiation. It is not necessary in every skin characterization, but in many cases, it can be very useful to represent the optical properties by a model with fewer unknown parameters. In contrast, the latter which describes how the physical structure of skin is perceived by THz waves, is essential to convert the THz field information to skin-related parameters. Whether to combine a skin model with a dielectric model or not is a trade-off problem: between the model accuracy and result accuracy, and there is a large divergence between different approaches. For example, establishing a comprehensive and precise model of skin may provide an accurate description about the light-skin interaction, but may result in too many unknown parameters that cannot be solved unambiguously. Combining various models to simplify this may reduce the credibility of the results, as errors in each model could be propagated and summed up. Therefore, a comprehensive overview and discussion of these models is necessary for the advancement of THz in vivo studies of skin. 

### 2.1. Dielectric Model

#### 2.1.1. Double Debye Model

The double Debye model is truncated from the Debye model and was first introduced to model the permittivity of liquid water. The permittivity can be described by two relaxation terms: the slow relaxation and fast relaxation, which is usually accurate for describing water dielectric behavior below 2 THz. Based on the high water content in tissues and molecular interaction between water inside skin and THz radiation, the model is then used to predict the THz dielectric response of human skin. The double Debye model is generally written in the following form:

(1) $${\tilde{\varepsilon }}_{r}\left(w\right)=\varepsilon_{\infty }+\frac{\varepsilon_{s}-\varepsilon_{2}}{1+jw\tau_{1}}+\frac{\varepsilon_{2}-\varepsilon_{\infty }}{1+jw\tau_{2}}$$

where $\varepsilon_{\infty }$ is the limiting permittivity at high frequencies and $\varepsilon_{s}$ is the static permittivity at low frequencies, $\varepsilon_{2}$ is the transitional permittivity at intermediate frequencies and $\tau_{1}$,$\tau_{2}$ correspond to the relaxation time of the slow and fast relaxation processes when the hydrogen bonds break and reorient and move to a new tetrahedral site, respectively. $\varepsilon_{\infty }$, $\varepsilon_{s}$, $\varepsilon_{2}$, $\tau_{1}$ and $\tau_{2}$ are the five double Debye parameters. Here are listed published values from reference [41] for the five parameters of water and skin: $\varepsilon_{s}^{water}=78.8$, $\varepsilon_{2}^{water}=6.6$, $\varepsilon_{\infty }^{water}=4.1$, $\tau_{1}^{water}=10.6$, $\tau_{2}^{water}=0.18$ and $\varepsilon_{s}^{skin}=60.0$, $\varepsilon_{2}^{skin}=3.6$, $\varepsilon_{\infty }^{skin}=3$, $\tau_{1}^{skin}=10.0$, $\tau_{2}^{skin}=0.2$. The comprehensive summary of values of double Debye parameters for water and different tissues can also be found in reference [42].

**Figure 2:**
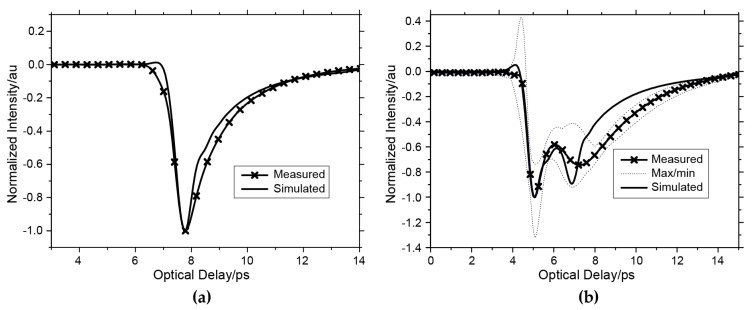
Comparison of measured and simulated impulse functions of the (**a**) (volar) forearm and (**b**) the palm. Reprinted with permission from ref. [29]. Copyright 2004 Physics in Medicine and Biology.

Pickwell et al. demonstrated that human skin can be modeled using the double Debye model and further employed finite difference-time-domain (FDTD) techniques to simulate the THz response of human forearm and palm tissues [29,41]. A Gaussian filter function is applied to the ratio of the sample to air reference to remove the low and high frequencies noise components to extract the suitable THz wave response which is called the impulse function. As shown in Figure 2, the simulated pulses fit the measured volar forearm and human palm impulse functions with high correlation coefficients. Truong et al. further employed this model for non-melanoma skin cancer classification, observing a high correlation between the model parameters and skin cancer [43]. However, the current procedure used to extract the double Debye parameters is not optimal and does not always yield consistent results. When the frequency goes up to 2 THz, it has been suggested that additional terms for resonant Lorentzian processes should be added to the model [1]. However, this brings more complexity for the extraction of the parameters. Most THz measurements of biological tissues are limited to being up to 2 THz. This is due to the attenuation increasing with frequency, as well as the limited dynamic range of the THz source. However, it is likely that the optimal frequency for THz sensing of tissue will be around 300 GHz to 1 THz due to the high sensitivity to water in this region (strong resonances due to hydrogen-bond network at 20 GHz and around 1 THz [44]). While higher frequencies will enable higher diffraction limited resolution to be achieved, for lower THz frequencies of interest, techniques such as single pixel THz cameras which employ spatial light modulation using photo-modulation [45] can be combined with THz imaging to achieve higher resolution (limited by the optics). In addition, as the double Debye model was originally used for water [1], its adaptability to tissues of a low water content, such as the SC in dry skin, remains questionable. This is because the non-water component may not follow double Debye relaxation characteristics, yet contributes significantly to the dielectric property of the composite. 

#### 2.1.2. Effective Medium Theory

THz dielectric interaction with a composite system consisting of components on subwavelength scales can be modeled by an effective medium theory (EMT), which enables the calculation of the composite permittivity from the permittivity of each individual component. Widespread research has employed this theory to estimate the water content of plants, biological phantoms and tissues [46,47]. A comprehensive review of various EMTs used in the THz regime, including their applicable situations and limitations, has been given by Scheller et al. [48]. To address the main idea of these applications, we first introduce the Bruggeman and Landau, Lifshitz, Looyenga (LLL) models, which are the most commonly used effective medium theories for THz biological applications. Taking the Bruggeman model as an example, the following equation shows the Bruggeman model applied to skin by considering the medium’s components as spheres embedded in water,

(2) $$\overset{n}{\underset{k=1}{\sum }}p_{k}\frac{\varepsilon_{k}-\varepsilon_{eff}}{\varepsilon_{k}+2\varepsilon_{eff}}=0,\;\left(\overset{n}{\underset{k=1}{\sum }}p_{k}=1\right)$$

where $p_{k}$ and $\varepsilon_{k}$ are the volume percentage and permittivity of the *k* th component and $\varepsilon_{eff}$ is the effective permittivity of the composite system. In these models, skin is treated as a binary composite of water and biological background, which is often assumed to have the same properties as dehydrated skin. The permittivity of water is usually treated as a known value as it has been accurately measured by many groups, and the permittivity of the biological background is estimated or measured by experiment. Fitting to the experimental results of skin is usually done by varying the water content in the model, with the best fit giving the estimated water percentage. Bennett et al. utilized this model to extract the water profile inside human skin which we will discuss further in the model with depth-dependent water concentration section.

The LLL model is another widely used EMT, which considers the particle shape of the biological background to be arbitrary. The following equation shows the LLL model:

(3) $$\sqrt[{3}]{\varepsilon_{eff}}=\overset{n}{\underset{k=1}{\sum }}p_{k}\sqrt[{3}]{\varepsilon_{k}},\;\left(\overset{n}{\underset{k=1}{\sum }}p_{k}=1\right)$$

**Figure 3:**
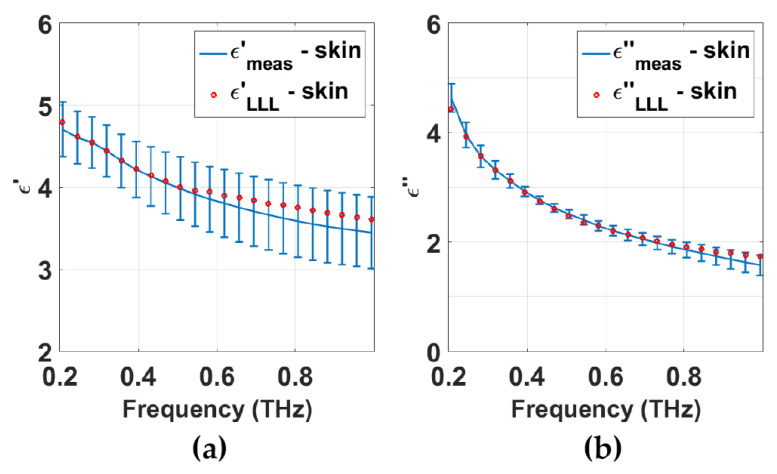
(**a**) Real part and (**b**) imaginary part of porcine skin permittivity. Reprinted with permission from ref. [47] Copyright 2017 Physics in Medicine and Biology.

The parameters required to calculate the effective permittivity in the LLL model are the same as those in the Bruggeman model, including the permittivity and volume fraction of each included component. He et al. measured dehydrated tissue samples including muscle, fat and skin and successfully employed the LLL model to estimate the water content in different types of porcine tissue ex vivo and the fitted water volumes agree well with previous values in literature [47]. Figure 3a,b show their measured and fitted permittivity of skin tissue. The high-degree match demonstrates that the permittivity of hydrated porcine skin can be well described by the LLL model. 

Hernandez-Cardoso et al. applied THz screening for early diagnosis of diabetic foot syndrome based on changes in the water content extracted by the LLL model [49]. They found that the water content extracted from THz measurements of subjects with diabetic foot syndrome is significantly lower than that of healthy subjects and together with quantitative analysis of 33 subjects, significant differences between the hydration of the feet of subjects in the control and the diabetic groups were found.

**Figure 4:**
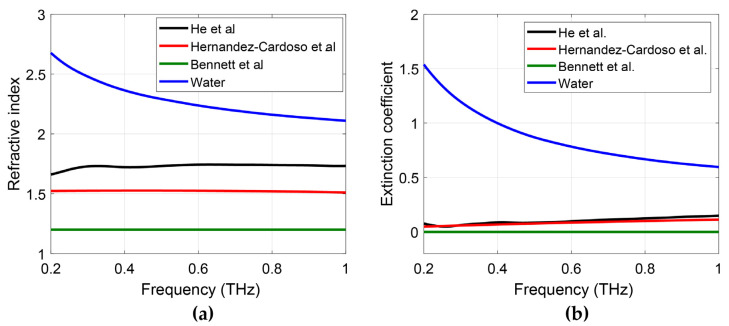
(**a**) Refractive index and (**b**) extinction coefficient of dehydrated skin obtained from work done by He et al. [47], Hernandez-Cardoso et al. [49] and Bennett et al. [51] and compared to that of water. Reprinted with permission from ref. [47]. Copyright 2017 Scientific Reports. Reprinted with permission from ref. [51]. Copyright 2011 IEEE Sensors Journal.

A comprehensive review of various EMTs used in the THz regime, including their applicable situations and limitations, has been given by Scheller et al. [48] and a specific investigation into the applicability of these different theories for biological tissues has been performed by Hernandez-Cardoso et al. [50] Despite the well-proved adaptability of EMTs to biological tissues, difficulties and errors remain when applying EMTs to in vivo skin characterizations. The first difficulty comes from the permittivity used for the dehydrated component, which is crucial in determining the final water concentration extracted from the fit [47,49,51]. It is nearly impossible to always measure the dehydrated properties of skin or each individual tissue layer for every in vivo measurement, thus the values are normally acquired from literature, either approximated from porcine skin or human skin. In doing this, two errors are introduced, with one from the deviation between the used values and the actual properties, and second from the large variation of the values in the literature. Different measured results from references [47,49,51] for the refractive indices and extinction coefficients of dehydrated skin are plotted and compared to that of water in Figure 4. As shown by the figure, the refractive index of dehydrated skin is less frequency-dependent than that of water, which also indicates that the frequency dependence of skin mainly comes from the large component of water in the skin. The difference in the measured properties of dehydrated skin mainly comes from the skin types and dehydration processes used, as reference [49] used human biopsies while the other two [47,51] used porcine skin to mimic human skin. Apart from the accuracy of the biological background, another error in applying EMTs to in vivo skin characterization is the questionable validity of these theories for this application. Various factors could invalidate the use of EMTs, e.g., the roughness of the skin [52] and the potential anisotropy in the SC [53] could break the assumption of both the Bruggeman and LLL EMTs that the included particles should be subwavelength and homogeneously distributed. The different properties between free water and bound water may also affect the extracted water content from the fit [54]. Therefore, the use of EMTs and the accuracy of the results obtained should be estimated by taking these factors into consideration.

### 2.2. Structural Models for THz Waves

Biologically, skin contains three main layers: stratum corneum (SC), epidermis, and dermis layers, with thicknesses that vary with location on the body and from person to person. Skin is primarily composed of water, which makes up about 20% to 70% of the skin, other components include collagen, elastin and other proteins [53,55]. THz waves can probe the structure of skin such as the SC and epidermis based on the clear differences between the two layers. As determined by confocal Raman spectroscopy, the SC has a depth-dependent water concentration gradient and the epidermis has a more constant value [56]. Therefore, models further separating the SC into multiple layers of different water fractions have been proposed. Based on different assumptions of water gradient changes inside skin, several structural models were proposed with EMTs to relate the water gradient with optical indices. However, the layered cellular structure originating from the flattened corneocytes in the SC induces anisotropy which is polarization sensitive and can also be probed in the THz regime. Next, we overview four structural models reported in the literature.

#### 2.2.1. Single Layer Model

From the water-concentration point of view, it is necessary to at least separate the skin into a SC-epidermis two-layer structure, as the SC is usually less hydrated compared to the inner epidermis. Nevertheless, treating the skin as a homogeneous semi-infinite single layer has been widely applied [28,38,39], due to the simplicity in characterizing the THz optical properties of skin. In this case, a dielectric model is unnecessary, as the complex permittivity can be solved by the analytical solution of the transfer function, or by numerically minimizing the difference between the theoretical and experimental transfer functions. For example, when s-polarization is used in a window-based reflection setup, the transfer function $\tilde{H}$, that is, the complex spectral ratio between the sample and reference reflections, can be expressed using Fresnel’s equations and Snell’s Law, as shown in Equations (4) and (5) respectively [27,38,39]

(4) $$\tilde{H}=\frac{r_{qs}}{r_{qa}}=\frac{{\tilde{n}}_{q}\cos\theta_{q}-{\tilde{n}}_{s}\cos\theta_{s}}{{\tilde{n}}_{q}\cos\theta_{q}+{\tilde{n}}_{s}\cos\theta_{s}}\cdot \frac{{\tilde{n}}_{q}\cos\theta_{q}+{\tilde{n}}_{a}\cos\theta_{a}}{{\tilde{n}}_{q}\cos\theta_{q}-{\tilde{n}}_{a}\cos\theta_{a}}$$

(5) $${\tilde{n}}_{q}\sin\theta_{q}={\tilde{n}}_{a}\sin\theta_{a}={\tilde{n}}_{s}\sin\theta_{s}$$

where $\tilde{n}$_q_ (*θ*_q_), $\tilde{n}$_a_ (*θ*_a_), $\tilde{n}$_s_(*θ*_s_), are the complex refractive indices (incident angles) of quartz, air and skin, respectively. In this case, the analytical solution of the complex refractive index of skin can be expressed as:

(6) $${\tilde{n}}_{s}=\sqrt{X^{2}+{\tilde{n}}_{q}{}^{2}sin^{2}\theta_{q}}$$

where

(7) $$X=\frac{\left(1+\tilde{H}\right){\tilde{n}}_{a}{\tilde{n}}_{q}\cos\theta_{a}\cos\theta_{q}+\left(1-\tilde{H}\right){\tilde{n}}_{q}{}^{2}cos^{2}\theta_{q}}{\left(1+\tilde{H}\right){\tilde{n}}_{q}\cos\theta_{q}+\left(1-\tilde{H}\right){\tilde{n}}_{a}cos\theta_{a}}$$

In other geometries where the analytical solution is complicated or unavailable, a numerical optimization algorithm can be used to find the solution. Even in this case, the solution can normally be easily found because the solution at each frequency ${\tilde{n}}_{s}\left(\omega \right)$ is independently extracted from the corresponding transfer function $\tilde{H}\left(\omega \right)$, which is a two-parameter optimization problem. The merit of using this model is that the unique solution can be easily found without a need for any dielectric model to simplify. However, the clear limitation is that it omits the boundary between the SC and epidermis. The complex refractive index found in this model neither represents the SC, the epidermis, nor the effective combination of them. Therefore, results from this model are only comparable if they are from setups that are completely the same (same incident angle, medium and polarization).

#### 2.2.2. Two-Layer Model

**Figure 5:**
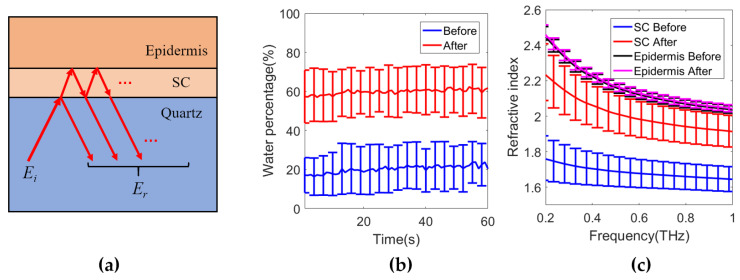
(**a**) Diagram of the THz interaction with a two-layer skin model consisting of SC and epidermis; (**b**) Water content in the SC and (**c**) refractive index in the SC and epidermis, before and after the application of silicone gel sheeting, respectively [35].

A two-layer skin model obtained by separating the skin into the SC and epidermis layers is much more reasonable in terms of their water concentrations. The most obvious problem induced however, is the characterization difficulty. In this case, the transfer function is now a function of the optical properties of the two layers (if the thickness of the SC $d_{SC}$ is assumed as known a priori), $\tilde{H}\left(\omega \right)=f\left[{\tilde{n}}_{SC}\left(\omega \right),{\tilde{n}}_{Ep}\left(\omega \right)\right]$, where ${\tilde{n}}_{SC}\left(\omega \right)$ and ${\tilde{n}}_{Ep}\left(\omega \right)$ are the complex refractive indices of the SC and epidermis. The number of unknown parameters is more than the number of known values. Hence, no unique solution can be found. A commonly used approach to solve this is using a EMT to describe the dielectric properties of each layer, such that the complex refractive indices of each layer, which contains multiple unknown complex values in the frequency-domain, is simply represented by a fitting parameter, that is the water content. Wang et al. measured the effect of silicone gel sheeting on normal skin and treated skin as a two-layer structure by assuming that the water content in the epidermis and dermis layers are very similar [35]. They measured 10 subjects before and after applying the silicone gel sheeting for 1 min with the experimental setup the same as that shown in Figure 1. By applying an LLL EMT (Equation (3)), the permittivity and refractive index in each layer is obtained as a function of water concentration as shown in Equation (8). Illustrated in Figure 5a, by applying the Fresnel equations, the reflection coefficient of the three-layer structure: quartz-SC-epidermis is calculated, shown in Equation (9):

(8) $${\tilde{n}}_{i}=\sqrt{{\tilde{\varepsilon }}_{eff,\;i}}=f\left(p_{i}\right)$$

(9) $$r_{\mathrm{qse}}=\frac{r_{qs}+r_{se}\times \exp(-i2\beta )}{1+r_{qs}r_{se}\times \exp(-i2\beta )}$$

where $p_{i}$ is the water volume percentage in the *i* th layer and *r_qs_, r_se_* are the reflection coefficients of the quartz-SC and SC-epidermis interfaces calculated by Equations (10) and (11). *β* is given by Equation (12). Note that $\tilde{n}$*_q_*, $\tilde{n}$*_s_*, $\tilde{n}$*_e_* and *θ_q_*, *θ_s_*, *θ_e_* are the complex refractive index and incident angle in quartz, SC and epidermis respectively and *d_s_* is the thickness of the SC. The incident angle in each layer *θ* is related to the complex refractive indices of each layer by Snell’s law given in Equation (13). Divided by the reflection from the quartz-air interface shown in Equation (14), the complex reflection ratio is obtained as illustrated by Equation (15) as a function of water content ($p_{i})$ in each layer and SC thickness(*d_s_*) which is then fitted to the measured data in the frequency domain.

(10) $$r_{qs}=\frac{{\tilde{n}}_{q}\cos\theta_{q}-{\tilde{n}}_{s}\cos\theta_{s}}{{\tilde{n}}_{q}\cos\theta_{q}+{\tilde{n}}_{s}\cos\theta_{s}}$$

(11) $$r_{se}=\frac{{\tilde{n}}_{s}\cos\theta_{s}-{\tilde{n}}_{e}\cos\theta_{e}}{{\tilde{n}}_{s}\cos\theta_{s}+{\tilde{n}}_{e}\cos\theta_{e}}$$

(12) $$\beta =\frac{2\pi d_{s}{\tilde{n}}_{s}cos\theta_{s}}{\lambda }$$

(13) $${\tilde{n}}_{a}\sin\theta_{a}={\tilde{n}}_{q}\sin\theta_{q}={\tilde{n}}_{s}\sin\theta_{s}={\tilde{n}}_{e}\sin\theta_{e}$$

(14) $$r_{qa}=\frac{{\tilde{n}}_{q}\cos\theta_{q}-{\tilde{n}}_{a}\cos\theta_{a}}{{\tilde{n}}_{q}\cos\theta_{q}+{\tilde{n}}_{a}\cos\theta_{a}}$$

(15) $${\tilde{H}}_{cal}=\frac{r_{qse}}{r_{qa}}=f\left(p_{i},d_{s}\right)$$

Figure 5b shows the fitted water concentration in the SC and (c) shows the refractive index in each layer before and after the application of silicone gel sheeting respectively. The water percentage in Figure 5b is shown as a function of occlusion time, which slightly increases throughout the measurement of the skin before the application of the silicone gel sheeting due to the water accumulation in the SC caused by occlusion of the skin by the imaging window. The water percentage in the SC increased from around 20% to 60% before and after applying silicone gel sheeting whereas the fitted water concentration in the epidermis (not shown) did not vary much and only changed from 75.7 ± 2.9% to 76.9 ± 2.5%. This is also shown by the refractive index of the epidermis plotted in Figure 5c. The refractive index of the SC increased significantly before and after the application of silicone gel sheeting. The results show that combining the two-layer skin model with the EMT is able to individually extract the properties of the SC and the epidermis, demonstrating the ability of THz waves to sensitively probe the hydration level of different skin layers.

#### 2.2.3. Model with Depth-Dependent Water Concentration

**Figure 6:**
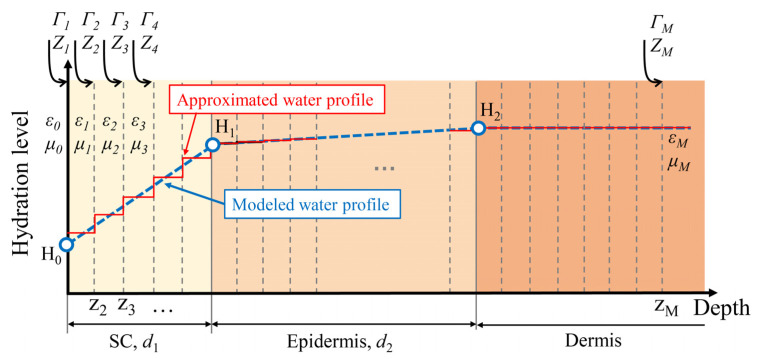
Schematic diagram showing the water profile in skin and the derivation of the reflection of a plane wave by a slab of stratified permittivity and permeability. The blue curve is the modeled water profile defined by *H*_0_, *H*_1_, *H*_2_ and *d*_1_*, d*_2_. The red curve is the approximated water profile used to estimate the permittivity in each layer.

- Stratified media model:

A more comprehensive THz structure model is one which considers the water concentration variation with skin depth, especially in the SC. The water concentration change with depth is usually observed by confocal Raman spectroscopy [57,58], which is also a technique frequently used to determine the SC thickness by identifying the point where the water concentration starts to become less depth-dependent. Bennett et al. employed the stratified media model to extract the water profile inside skin by assuming the water concentration in the SC and epidermis follows a linear function with depth while the water concentration in the dermis is constant [51], as shown by the blue dashed line in Figure 6. Therefore, the water gradient inside the skin can be represented by the thickness of the SC (*d*_1_) and epidermis (*d*_2_), and the hydration level at the SC surface (*H*_0_), SC-epidermis boundary (*H*_1_) and epidermis-dermis boundary (*H*_2_). There is no direct equation to describe the light-skin interaction which has a linear depth-dependent water concentration. Instead, it is separated into multiple discrete layers to approximate the continuous water changes as shown by the red curve in Figure 6. With the number of slabs increasing, the accuracy increases as well as the computational complexity. However, considering that the wavelength is of the order of hundreds of micrometers, a slab thickness of 1 μm is sufficient to represent the water variation that can be sensed by the THz waves. A larger slab thickness can be used if the water-variation slope is flat, such as that in the epidermis. Given that the penetration depth is usually less than 100 μm, only a few tens of layers are needed in total, thus the computation time is usually considerably short. The permittivity of each layer ($\varepsilon_{m}$) is determined by water concentration ($p_{i}$) with the Bruggeman EMT, which can be expressed as Equation (16).

(16) $$\varepsilon_{m}=f\left(p_{i}\right)$$

**Figure 7:**
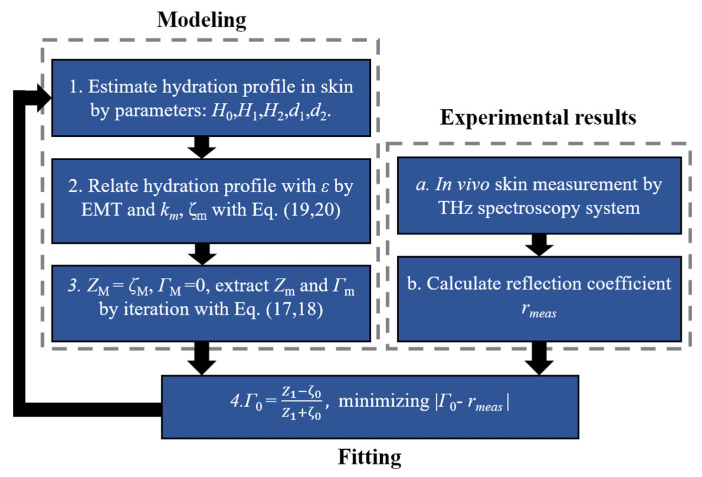
Flowchart for extracting skin hydration by Stratified media model.

Together with the incident angle *θ*, we can represent the impedance (*Z_m_*) and the reflection coefficient *Γ_m_* in layer *m* by Equations (17) and (18), where the longitudinal propagation constant (*k_m_*) and characteristic impedance (*ζ*_m_) are related to the permittivity $\left(\varepsilon_{m}\right)$ and permeability $\left(\mu_{m}\right)$ of each layer by Equations (19) and (20). Details can be found in reference [51]. The thickness and depth at the corresponding layer are given by *t_m_* and *z_m_* respectively. Therefore, the water gradient inside the skin, eventually represented by *H*_0_, *H*_1_, *H*_2_ and *d*_1_, *d*_2_, determines the reflection coefficient at the skin surface. In addition, this work employed a non-contact measurement and Rayleigh scattering due to the roughness of the skin surface is calibrated. By fitting to the measured reflection, the water gradient can be calculated. To better illustrate the process of calculating the refection coefficient, Figure 7 shows the flowchart of the procedure.

(17) $$Z_{m}=\zeta_{m}^{te}\frac{Z_{m+1}+j\zeta_{m}^{te}\tan(k_{m}t_{m})}{\zeta_{m}^{te}+jZ_{m+1}\tan(k_{m}t_{m})}$$

(18) $$\Gamma_{m}=\frac{Z_{m+1}-\zeta_{m}^{te}}{Z_{m+1}+\zeta_{m}^{te}}e^{-2jk_{m}z_{m+1}}$$

(19) $$k_{m}=w\sqrt{\varepsilon_{0}\mu_{0}}\sqrt{\varepsilon_{m}-sin^{2}\theta }$$

(20) $$\zeta_{m}^{te}=\frac{w\mu_{m}}{k_{m}}$$

**Figure 8:**
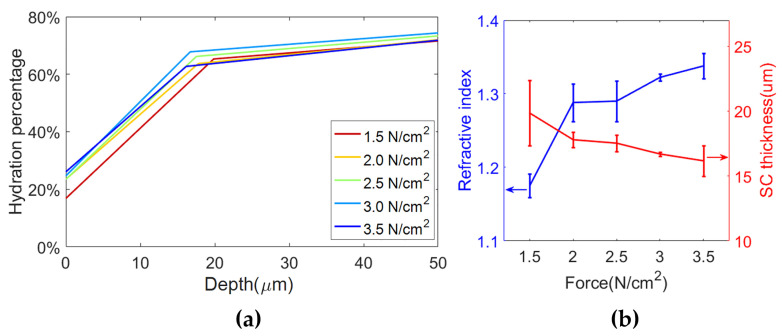
(**a**) Water profile in skin under different applied pressures; (**b**) Refractive index of dehydrated skin and SC thickness under different pressures. The error bars in (**b**) are the standard deviation of three measurements [38].

Wang et al. utilized this model to study the effects of pressure during THz in vivo measurements [51]. They measured human volar forearms with target pressures ranging from 1.5 N/cm^2^ to 3.5 N/cm^2^ in a reflection geometry with pressure sensors to give real-time feedback. A quartz window was employed to flatten the skin. The reflected THz signal and skin optical properties changed under different pressures. Instead of using a constant value for the refractive index of dehydrated skin in the EMT model, they treated the refractive index of dehydrated skin as another fitting parameter. After fitting the data measured under different pressures with the stratified media model, the water profiles and the refractive index of dehydrated skin are extracted as shown by Figure 8. It is found that increasing the applied pressure leads to an increase in the hydration level of the SC surface as shown by Figure 8a. It also shows that the SC thickness decreased slightly with increased pressures, which is shown by the red curve in Figure 8b. As a result of the increased hydration level, the increase in the contact pressure also leads to an increase in the refractive index of dehydrated skin, as shown in Figure 8b. This can be explained by the fact that, at low contact pressures, there is the inevitable subtle air gap between the quartz window and skin due to the texture of skin.

**Figure 9:**
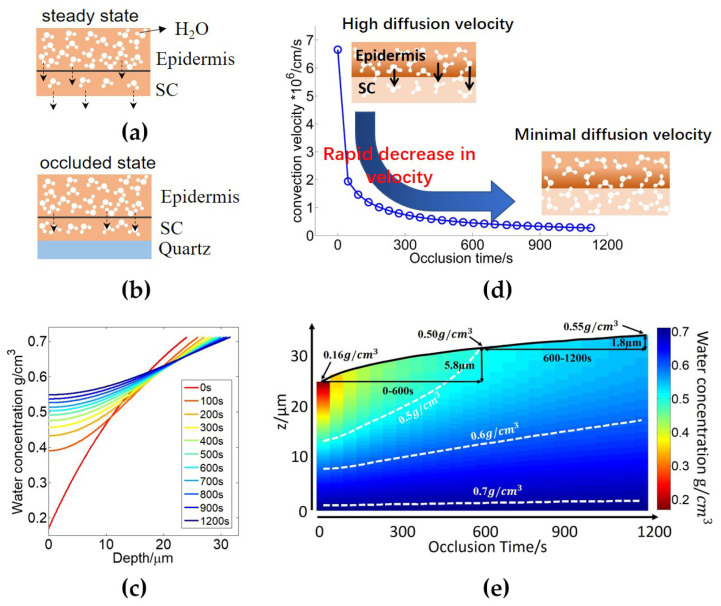
(**a**) Water dynamics of skin in a steady state; (**b**) Water dynamics of skin under occlusion; (**c**) The water concentration profile at different occlusion times; (**d**) The convection velocity as a function of the occlusion time; (**e**) A colormap of the water concentration as a function of depth (z) in skin and occlusion time. The black curve shows the change in the thickness of the SC with occlusion time. The white dashed curves are the contour lines of the water concentration at 0.5, 0.6 and 0.7 g/cm^3^. Reprinted with permission from ref. [27] Copyright 2019 Journal of Biophotonics.

Sun et al. further developed the stratified media model by combining it with the SC swelling model to estimate water diffusion and the hydration profile inside the skin when skin is occluded by a quartz window. The experimental setup used is the same as that shown in Figure 1. As shown in Figure 9a,b, when skin is in a steady state, water in the epidermis and SC can diffuse to the surroundings. When the imaging window prevents water in the SC diffusing to the surroundings, water accumulates in the SC causing the SC to increase in hydration and swell, which is called the occluded state. Instead of approximating water concentration as a linear function with depth in the SC, they used convection and diffusion equations to describe the water concentration $W\left(z,t\right)(g/cm^{3}$) as a function of depth *z* (cm) and time *t* (s). Its relationship with the water diffusion coefficient D(t) is given by Equation (21),

(21) $$D\left(t\right)\frac{\partial^{2}W\left(z,t\right)}{\partial z^{2}}-u\left(t\right)\frac{\partial W\left(z,t\right)}{\partial z}=\frac{\partial W\left(z,t\right)}{\partial t}$$

$D\left(t\right)(cm^{2}/s$) is assumed to take the form of an exponential function (Equation (22)), to be determined by fitting to the measured THz signal during 20 min of occlusion. u(t)(cm/s) is the water convection velocity which determines the speed of SC swelling. There are several assumptions made about the skin during the occlusion process which yield the boundary conditions Equations (23)–(25), these are explained in detail here. When skin is at the steady state, the water profile in the SC does not change with time. This gives the first boundary condition by the initial water concentration in the SC at time 0, expressed by Equation (23), where *W_S0_* ($g/cm^{3}$) and *W_B0_* ($g/cm^{3}$) are the initial water concentrations at the surface and bottom of the SC respectively. *L*_0_ is the initial SC thickness. They can be found by fitting to the measured data when skin is first put onto the quartz window. At the occluded state, the water flux on the surface of the skin decreases to zero while the water concentration at the SC-epidermis boundary (*z* = 0) remains unchanged. This leads to the second boundary conditions in Equation (24). With the convection velocity (*u*) equal to the swelling velocity of the SC, the third boundary condition can be given by Equation (25). The finite difference method [59] was employed to calculate the numerical solution of Equations (21)–(25). Details of the fitting and procedure diagram can be found in reference [27].

(22) $$D=D_{0}e^{\alpha \left(W\left(0,t\right)-W\left(L\left(t\right),t\right)\right)},\;D_{0},\alpha \geq 0$$

(23) $$W\left(z,0\right)=1+\left(W_{B0}-1\right){\left(\frac{W_{S0}-1}{W_{B0}-1}\right)}^{\frac{z}{L_{0}}}$$

(24) $$\{\begin{array}{l} {-D\left(t\right)\frac{\partial W\left(z,t\right)}{\partial z}|}_{z=L\left(t\right)}=0\\ W\left(0,t\right)=W_{B0} \end{array}$$

(25) $$u\left(t\right)=\frac{dL\left(t\right)}{dt}=\frac{D\left(t\right)}{W_{B0}-1}{\frac{\partial W\left(z,t\right)}{\partial z}|}_{z=0}$$

As shown by Figure 9c, the water concentration in the SC increases with skin depth at different occlusion times and as the occlusion time increases. The SC surface becomes more hydrated. At the early stages of occlusion, hydration in the SC increases faster than at the later stages. The convection velocity is positive and decreases with occlusion time as illustrated in Figure 9d which means that during occlusion, the SC thickness increases, however, the rate of expansion decreases with occlusion time. Figure 9e better reveals this phenomenon and shows how the water distribution in the SC changes with occlusion time. During the occlusion process, the SC surface hydration changes from 0.16 g/cm^3^ to 0.55 g/cm^3^ and SC swells by approximately 7.6 μm. However, the hydration and thickness of the SC increase most dramatically at the onset of occlusion compared to later stages of occlusion due to the decreasing water concentration gradient in the SC. This work estimates the water diffusivity in occluded skin for the first time. However, various assumptions were made to enable the model to be solved, such as the zero water flux at the skin surface, equaling the convection velocity to the swelling velocity, the constant water profile at the steady state, and the exponential diffusivity function, etc.

**Figure 10:**
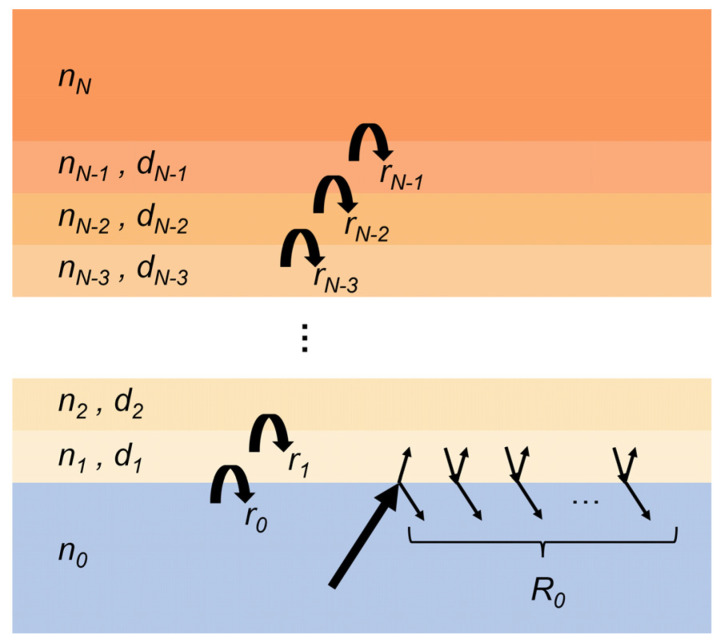
Schematic diagram of the multiple layer structure and the derivation of the reflection coefficient based on the Fresnel equations. *n_m_* (*m* > 0) is the refractive index of the *m*th layer calculated using an EMT. *d_m_* is the thickness of the *m*th layer. *r_m_* is the reflection coefficient from medium *m* to *m* + 1. *R*_0_ is the reflection of light incident from medium 0 to the N-layer structure.

- Fresnel equation-based model:

It is worth mentioning that an alternative way to describe the multilayer structure of skin is using the Fresnel equations. The Fresnel reflection coefficient for a multiple layer structure is based on the Fresnel equation for a three-layer structure, which takes the form of Equation (9). Consider a multiple layer structure as illustrated in Figure 10 consisting of *N* + 1 layers with medium 0 and *N* being semi-infinite. Medium 0 is usually the window in a practical measurement. The *m*th layer (0 < m < *N*) has a thickness of $d_{m}$. The complex refractive indices of the skin layers are determined by its water concentration effectively calculated from the EMT. The reflection of a light illuminating from medium *N* − *j* (*N* ≥ *j* > 1) to the inner layers is denoted as $R_{N-j}$, which is expressed as Equation (26),

(26) $$R_{N-j}=\frac{r_{N-j}+R_{N-j+1}\times exp\left(-2i\beta_{N-j+1}\right)}{1+r_{N-j}R_{N-j+1}\times exp\left(-2i\beta_{N-j+1}\right)}$$

where $R_{N-j+1}$ represents the reflection from medium *N* − *j* + 1 to the inner layers until medium *N*, which is again calculated by Equation (26) if *N* − *j* + 1 < *N* − 1. *exp* means exponent. When *N* − *j* + 1 = *N* − 1 (i.e., *j* = 2), $R_{N-1}$ equals to $r_{N-1}$ and is calculated using the two-layer Fresnel reflection coefficients, depending on the angle of incidence and the polarization. $\beta_{N-j+1}$ is the propagation coefficient in medium *N* − *j* + 1 and takes the form of Equation (27),

(27) $$\beta_{N-j+1}=\frac{2\pi d_{N-j+1}}{\lambda }n_{N-j+1}cos\theta_{N-j+1}$$

where $\theta_{N-j+1}$ is the refraction angle in medium *N* − *j* + 1, relating to the incident angle in medium 0 by Snell’s Law, as given in Equation (5). $n_{N-j+1}$ and $d_{N-j+1}$ are the complex refractive index and thickness of medium *N* − *j* + 1 respectively, and λ is the wavelength of the light. Therefore, the reflection from medium 0 to the inner layers is calculated by Equation (26) with *j* = *N* and repetitively substituting Equation (26) to calculate the reflections from the inner layers.

**Figure 11:**
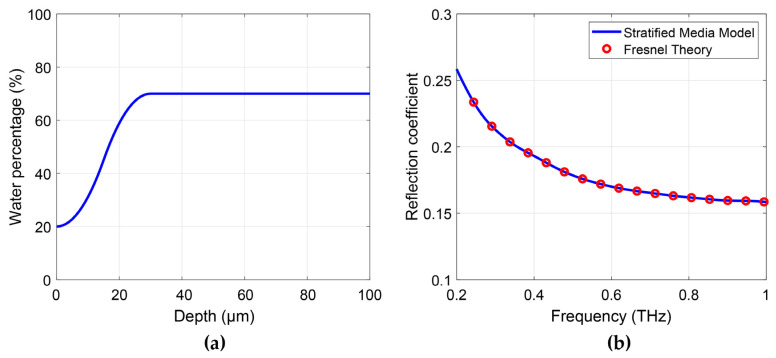
(**a**) Defined water concentration gradient; (**b**) Reflection coefficients calculated based on the stratified media model and Fresnel theory, respectively.

The multiple layer Fresnel equation is theoretically equivalent to the impedance method used in Bennett’s work [51]. To confirm this, we have calculated the reflection coefficient of a theoretical phantom with skin in contact with a quartz window based on the Fresnel equations and the stratified media model. The same water concentration gradient (Figure 11a) is used for both calculations. The results are shown in Figure 11b, with the Fresnel reflections shown as red open circles and the reflections calculated by the impedance method shown as the blue curve. The equivalent results confirm that both methods can be used for multiple layer structures.

In summary, the skin model with a depth-dependent water concentration is an extension of the two-layer model by further considering the water variation in the SC and epidermis. This is an important correction especially for the SC as the water concentration changes dramatically. Of course, it further complicates the model to introduce more variables that need to be determined, and usually more assumptions should be made to enable a global minimum when fitting to the experimental results. For example, in the stratified medium model, the water distribution is assumed to follow a linear relationship with depth such that the water fraction at each layer can be determined by just estimating the water fractions at the boundaries (i.e., *H*_0_, *H*_1_, and *H*_2_). Whether the fitting parameters can be found unambiguously depends on the number of parameters to be found, the sensitivity of the system, the noise of the measured data and the algorithm designed for the optimization. Therefore, there is a trade-off between the model accuracy and the result accuracy. A comprehensive and accurate model may not be able to be accurately solved, while adding more assumptions and simplifications may reduce the credibility of the results.

#### 2.2.4. Model with Anisotropic Stratum Corneum

**Figure 12:**
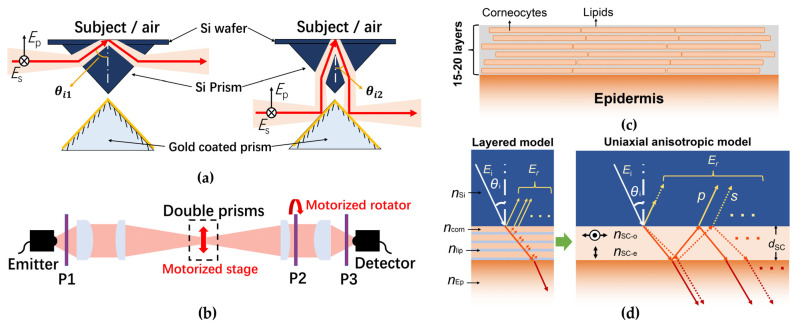
(**a**) Illustration of the double prism system and the two alternative THz optical paths; The double-prism system is assembled in (**b**) a THz transmission-form ellipsometer; (**c**) The “bricks and mortar” biological structure in the SC; (**d**) layered model (**left**) and the anisotropic skin model (**right**) [53].

Recently, Chen et al. explain a new hypothesis that birefringence exists in the SC due to the layered cellular structure. This was proven by measuring the skin using complementary ellipsometry configurations [53]. The SC is composed of flattened corneocytes (the SC cells) and a lipid matrix. The “bricks and mortar” structure of the SC is shown by Figure 12c. A composite made of layers with subwavelength thickness can be effectively equivalent to an anisotropic birefringent medium [60]. Therefore, the lamellar cellular structure leads to anisotropy in the SC, with its ordinary and extraordinary components taking the directions indicated in Figure 12d. The anisotropy invalidates the use of EMTs. In this case, the skin consists of three unknown optical components, being the complex refractive indices of the SC ordinary component ${\tilde{n}}_{SC-o}$, SC extraordinary component ${\tilde{n}}_{SC-e}$ and the epidermis ${\tilde{n}}_{Ep}$. The traditional reflection setup cannot provide enough spectral information to extract the skin properties and reveal the anisotropic properties of the SC. Thus, Chen et al. utilized a multi-configuration ellipsometer shown in Figure 12a,b to provide four sets of independent spectral information. This is achieved using a double right-angle prism system. One is made of Si, another is a gold coated prism which provides perfect reflection. The gold coated prism is symmetrically mounted under the Si prism. The double prism system is put onto the transmission geometry and by adjusting the prism system height, two optical paths can be achieved, as illustrated in Figure 12a. When the prism system is placed at the lower position, the THz beam is directly refracted by the upper Si prism to the skin with incident angle at the interface being $\theta_{i1}$. When the prism system is placed at the upper position, the THz beam is first reflected by the lower gold coated prism to alter the incident angle into the upper Si prism, with the incident angle at Si-skin interface being $\theta_{i2}$. The polarization of the THz beam is controlled by three polarizers, as shown in Figure 12b. P1 and P3 are fixed at 45° to the s- direction, and P2 is mounted on a rotator to adjust the s-/p- directions. In this way, the p- and s- reflections under the two incident angles are measured, providing four sets of independent spectral information.

The established model consists of three layers with the middle layer being uniaxial anisotropic, with the optical axis perpendicular to the layers. Different from the isotropic models of skin mentioned above, p- and s-reflection coefficients of the anisotropic skin model can be expressed using a tri-layer Fresnel model shown by Equations (28) and (29).

(28) $$r_{p}^{Si-Skin}=\frac{E_{rp}^{Si-skin}}{E_{ip}}=\frac{r_{p}^{Si-SC}+r_{p}^{SC-EP}\times exp\left(-2\beta_{p}\right)}{1+r_{p}^{Si-SC}r_{p}^{SC-EP}\times exp\left(-2\beta_{p}\right)}$$

(29) $$r_{s}^{Si-Skin}=\frac{E_{rs}^{Si-skin}}{E_{is}}=\frac{r_{s}^{Si-SC}+r_{s}^{SC-EP}\times exp\left(-2\beta_{s}\right)}{1+r_{s}^{Si-SC}r_{s}^{SC-EP}\times exp\left(-2\beta_{s}\right)}$$

where

(30) $$r_{p}^{Si-SC}=\frac{{\tilde{n}}_{SC-o}{\tilde{n}}_{SC-e}cos\theta_{i}-{\tilde{n}}_{Si}{\left({\tilde{n}}_{SC-e}^{2}-{\tilde{n}}_{Si}^{2}sin^{2}\theta_{i}\right)}^{1/2}}{{\tilde{n}}_{SC-o}{\tilde{n}}_{SC-e}cos\theta_{i}+{\tilde{n}}_{Si}{\left({\tilde{n}}_{SC-e}^{2}-{\tilde{n}}_{Si}^{2}sin^{2}\theta_{i}\right)}^{1/2}}$$

(31) $$r_{s}^{Si-SC}=\frac{{\tilde{n}}_{Si}cos\theta_{i}-{\left({\tilde{n}}_{SC-o}^{2}-{\tilde{n}}_{Si}^{2}sin^{2}\theta_{i}\right)}^{1/2}}{{\tilde{n}}_{Si}cos\theta_{i}+{\left({\tilde{n}}_{SC-o}^{2}-{\tilde{n}}_{Si}^{2}sin^{2}\theta_{i}\right)}^{1/2}}$$

(32) $$r_{p}^{SC-Ep}=\frac{{\tilde{n}}_{Ep}{\left({\tilde{n}}_{SC-e}^{2}-{\tilde{n}}_{Si}^{2}sin^{2}\theta_{i}\right)}^{1/2}-{\tilde{n}}_{SC-o}{\tilde{n}}_{SC-e}cos\theta_{Ep}}{{\tilde{n}}_{Ep}{\left({\tilde{n}}_{SC-e}^{2}-{\tilde{n}}_{Si}^{2}sin^{2}\theta_{i}\right)}^{1/2}+{\tilde{n}}_{SC-o}{\tilde{n}}_{SC-e}cos\theta_{Ep}}$$

(33) $$r_{s}^{SC-Ep}=\frac{{\left({\tilde{n}}_{SC-o}^{2}-{\tilde{n}}_{Si}^{2}sin^{2}\theta_{i}\right)}^{1/2}-{\tilde{n}}_{Ep}cos\theta_{Ep}}{{\left({\tilde{n}}_{SC-o}^{2}-{\tilde{n}}_{Si}^{2}sin^{2}\theta_{i}\right)}^{1/2}+{\tilde{n}}_{Ep}cos\theta_{Ep}}$$

(34) $$\beta_{p}=\frac{\omega d}{c}\left(\frac{{\tilde{n}}_{SC-o}}{{\tilde{n}}_{SC-e}}\right){\left({\tilde{n}}_{SC-e}^{2}-{\tilde{n}}_{Si}^{2}sin^{2}\theta_{i}\right)}^{1/2}$$

(35) $$\beta_{s}=\frac{\omega d}{c}{\left({\tilde{n}}_{SC-o}^{2}-{\tilde{n}}_{Si}^{2}sin^{2}\theta_{i}\right)}^{1/2}$$

Note that $\theta_{i}$ is the incident angle at the Si-SC interface and $\theta_{Ep}$ is the refraction angle in the epidermis. d is the SC thickness. c is the speed of light. $E_{ip}$ and $E_{is}$ are the p- and s-incident fields, as indicated in Figure 12d. The p-reflection is dependent on the refractive index of the SC in the ordinary and extraordinary directions, while the s-reflection is solely related to the refractive index in the ordinary direction. The theoretical reference reflections $r_{p}^{Si-air}=E_{rp}^{Si-air}/E_{ip}$ and $r_{s}^{Si-air}=E_{rs}^{Si-air}/E_{is}$ can be calculated using the two-layer Fresnel equations. The four theoretical spectral ratios under different incident angles and polarizations can thus be theoretically calculated. By minimizing the difference between the four theoretical calculations and the corresponding experimental data, ${\tilde{n}}_{SC-o}$, ${\tilde{n}}_{SC-e}$ and ${\tilde{n}}_{Ep}$ were extracted.

**Figure 13:**
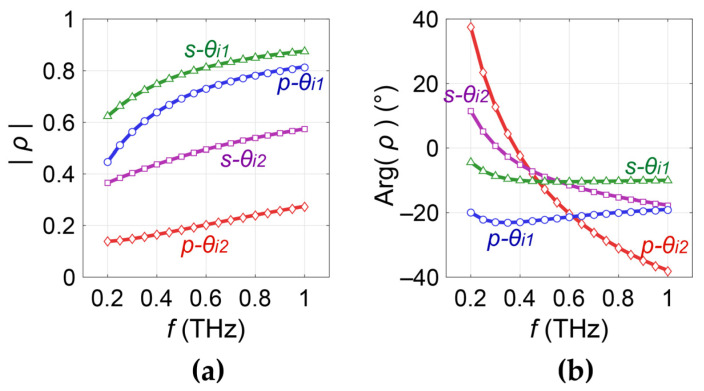
(**a**) The amplitude and (**b**) phase of the reflection ratios calculated for the four experimental geometries, indicated by *s-θ_i_*_1_, *p-θ_i_*_1_, *s-θ_i_*_2_, and *p-θ_i_*_2_. The symbols are the results from the layered model and the solid curves are the results from the anisotropic model [53].

To theoretically verify the birefringence induced by the lamellar SC cellular structure, the authors compared the reflection from a layered model made of a periodic corneocyte-lipid structure, and the reflection from the effective birefringent medium using the EMT for a lamellar-layer composite [53,60]. The reflections were divided by the Si-air reflections in the corresponding geometries to obtain the reflection ratios. The magnitude and phase of the ratios *ρ* for the layered and birefringent models under the four configurations mentioned above were calculated, shown as the symbols and solid curves in Figure 13a,b. The high-degree match for all the four curves proves that the layered model is physically identical to the anisotropic model. 

**Figure 14:**
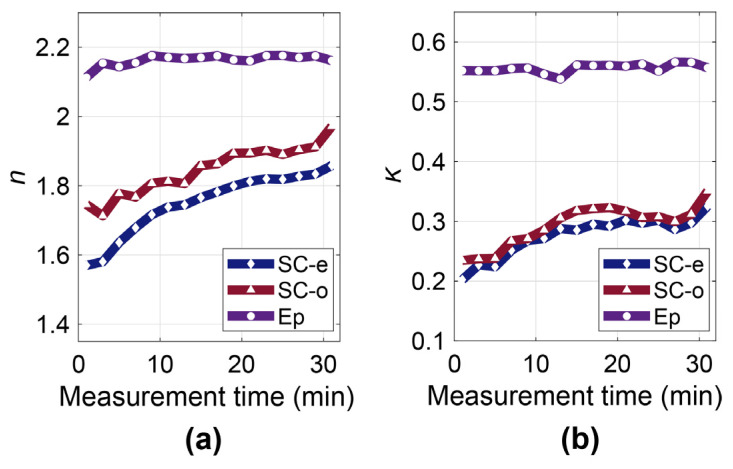
(**a**) The refractive index and (**b**) extinction coefficient of the SC in the extraordinary and ordinary directions and epidermis at 0.6 THz during 30 min of occlusion [53].

**Figure 15:**
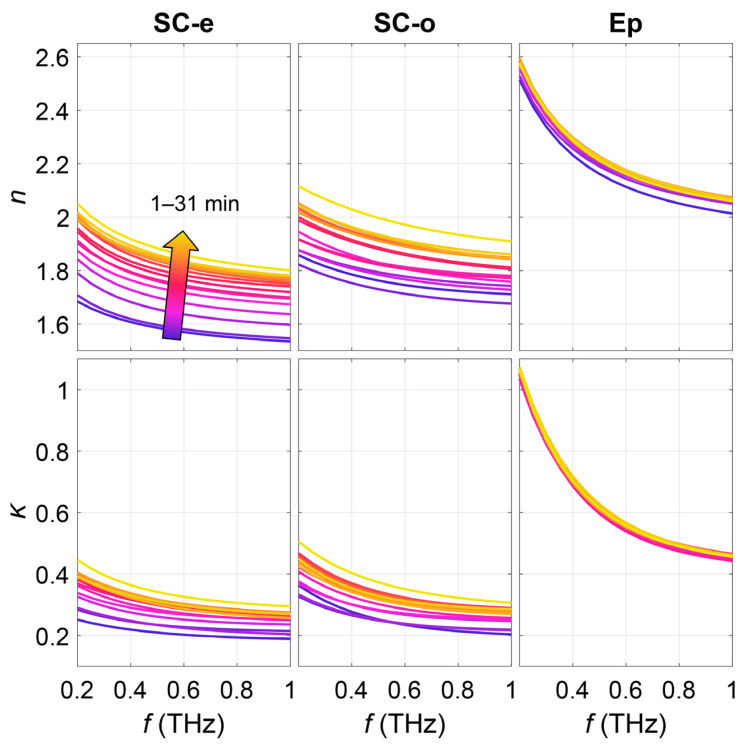
Refractive index and extinction coefficient spectra of the SC in the extraordinary and ordinary directions and epidermis during 30 min of occlusion [53].

They further experimentally verify the anisotropy of skin using the complementary ellipsometer. The volar forearm was measured under occlusion by the Si prism and fitted using the anisotropic skin model. During the measurement, subjects were asked to place their volar forearm on the Si prism for continuous 30 min. Figure 14a shows the change in the refractive index and extinction coefficient with occlusion time for one subject at 0.6 THz. Both the refractive index and extinction coefficient of the SC increase with occlusion time. While the refractive index and extinction coefficient of the epidermis remain almost unchanged. This proves that occlusion mainly changes the properties of the SC while the deeper layers of skin such as the epidermis are almost unaffected. Figure 15 shows the whole spectrum of the SC in the extraordinary and ordinary directions, showing that the time-dependency of the three skin components observed in Figure 14 also applies to the full spectrum from 0.2 to 1 THz. 

Usually, the refractive index and extinction coefficient are used to represent the hydration level inside tissues because water dominates these values. However, in the anisotropic skin models, both the water content and the structure affect the value of the refractive index and extinction coefficient. Therefore, dispersion and birefringence were used to represent the hydration level and anisotropic structure of skin, respectively. As mentioned above in Figure 4, optical properties of dehydrated skin can be considered achromatic, while water is highly dispersive. Therefore, the dispersion of skin is positively correlated with the hydration level of skin. Based on the fact that the refractive index of water decreases with frequency, dispersion is defined by the difference between first and last frequency point shown by Equation (36).

(36) $$\mathrm{Dispersion}=\left|{\tilde{n}}_{SC-e}\left(\omega_{1}\right)-{\tilde{n}}_{SC-e}\left(\omega_{m}\right)\right|$$

where ${\tilde{n}}_{SC-e}$ is the complex refractive index of skin in the extraordinary direction and $\omega_{1}$ and $\omega_{m}$ are 0.2 THz and 1 THz respectively. Birefringence is used to represent the structure and inhomogeneity of the SC. The birefringence of *n* is given by Equation (37).

(37) $$nBir=\overset{w_{m}}{\underset{w_{1}}{\sum }}[n_{SC-o}\left(\omega \right)-n_{SC-e}\left(\omega \right)]/m$$

**Figure 16:**
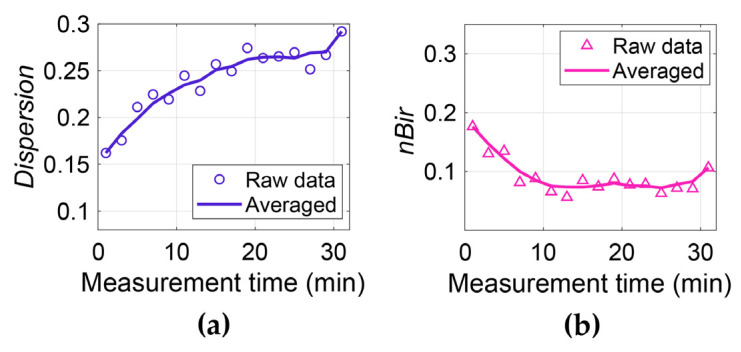
(**a**) Dispersion and (**b**) n birefringence of one subject. The symbols are the raw data and the solid curves are the moving average of every 5 data [53].

Figure 16 shows the dispersion and *nBir* of one subject, a clear increase in the dispersion with time is demonstrated which indicates the increased hydration of the SC. On the other hand, *nBir* decreases with time which means that the level of inhomogeneity decreases with occlusion time. The results show two variations in the SC, being the water accumulation and the reduced inhomogeneity mainly caused by the swelling of the SC which reduces skin furrows and roughness. This demonstrates the best advantage of using the complementary ellipsometer and the anisotropic model, that not just the hydration level of the skin can be detected, the SC structure can also be sensitively probed, providing valuable information for various skin studies and diagnosis. It should also be pointed out that in this case, the water-gradient in the SC is not directly extracted. Instead, it is reflected by the birefringence. The depth-dependent water concentration is a result of depth-dependent air compositions induced by the furrows and roughness, and it gives rise to the birefringence. Therefore, the model does not conflict with the model considering the water gradient, and it can be regarded as an improved model by further considering the anisotropy of the SC.

### 2.3. Comparison of Different Models

Table 1 summarizes and compares the dielectric and structure models applied for THz in vivo skin applications, highlighting their merits and limitations. 

In short, dielectric models include the double Debye model and EMT, with the former expressing the permittivity by its physical properties, and the latter regarding the investigated tissue as a composite of water and non-water substances. Therefore, the double Debye model is better at describing the physical characteristics of the tissue, represented by the double Debye parameters. It provides a better flexibility in classifying different tissues using these parameters, such as distinguishing cancerous and healthy regions [43]. It does not require a prior knowledge of the properties of the dehydrated tissue or water. The accuracy of the double Debye model remains questionable when the water concentration of a tissue is small, therefore its adaptability to dry tissues such as the SC requires further investigation. On the other hand, EMT is based on a prior knowledge about the properties of water and a biological background, with only the fraction of water needed to be determined from the fit. The obvious merit is therefore the very small number of fitting parameters needed. Thus, it is widely used in combination with multiple layer skin models. However, the biggest challenge comes from the biological background, which cannot always be measured specifically and there is a lack of a credible database as a reference. In addition, the EMT may become inaccurate when the assumptions of EMT cannot be satisfied, such as when the skin furrows and roughness introduce inhomogeneous air compositions in the SC.

As the accuracy of the four major structure models increases so does the complexity, there is also increasing difficulty in the characterization. The mathematical principle decides that only one set of complex refractive indices can be solved when only one spectral ratio is available. This applies to the single layer model to enable a straightforward solution to be found analytically or numerically. However, omitting the property difference between the SC and the epidermis is obviously a big error, thus the results extracted from this oversimplified model neither represent the SC nor the epidermis, giving no comparability between different measurement setups. The double-layer model makes an important correction to consider the reflection at the SC-epidermis boundary. As mentioned, it requires a proper dielectric model, usually an EMT, to reduce the number of fitting parameters. Results obtained from this model well display the water concentration difference between the SC and the epidermis, which further confirms the necessity of separating them in a structural model. Considering the depth-dependent water concentration makes the model better coincide with the water gradient observed by Confocal Raman Spectroscopy. In this case, multiple layers (>3) with discrete water fractions are needed to approximate the continuous water-concentration change. To reduce the number of unknown water fractions in these layers, usually a pre-defined water profile is needed, such as the linear model used in the stratified medium theory. However, attention should be given to verify the convergence of the fitting with the increased number of unknown parameters. Lastly, the anisotropic SC skin model was recently proposed by considering the birefringence caused by the lamellar SC cells. The depth-dependent water concentration is indirectly represented by the birefringence of the refractive index extracted. It is the most comprehensive model, without using any dielectric EMT to simplify it and thus provides a good credibility. However, the large number of unknown properties requires four complementary spectrums obtained from four uncorrelated geometries to provide a convergent optimization. 

## 3. Future Perspective

The variety of models and measurement protocols results in divergent results in different THz in vivo skin measurements, creating obstacles for comparisons between different studies. For example, as mentioned in Figure 4, by using different biological background refractive indices, the extracted water concentration can be different. Using a consistent measurement protocol is another important factor to ensure that results can be meaningfully compared [40]. Variables such as applied pressure and occlusion time should be carefully controlled as they significantly affect the reflectivity. Due to the occlusion effect rapidly changing the water concentration with time, current in vivo studies of skin are mostly point scans or line-scans [38,53]. By developing robust protocols, we can also overcome difficulties in comparing results taken on a variety of setups, such as with different angles of incident, polarizations, bandwidth. Faster, more accurate THz systems are needed before the skin models can be applied to interpret more complex applications such as drug diffusion along the vertical plane. Indeed, advances in single-pixel THz cameras are likely to pave the way for real applications [45]. Moreover, human skin is very diverse. Age, gender, and ethnicity could also be important factors that result in inconsistency. It has been reported that human skin of different ethnic types shows clear differences in structure and function [61]. For example, Asian skin in general shows higher water contents and higher SC lipid levels [61]. Studies have shown that aging skin shows decreased epidermis thickness [62], is more susceptible to become dry in low-humidity environments and is often characterized by roughness and wrinkling [63]. However, studies on the hydration levels in different genders do not show much relation. Gender differences have been investigated by Firooz et al., which showed slightly higher hydration in the female group but not statistically significant [64] while studies by Ehlers et al. [65] and Wilhelm et al. [66] showed no correlation between skin hydration and sex. These factors may also affect THz the THz response. Barker et al. demonstrated the clear difference in THz pulse for Asian male and Caucasian male skin [67]. Peralta et al. measured the THz optical differences during melanogenesis using in vitro skin models from Asian, Black, and Caucasian races [68]. However, there are still limited studies on the influence of different skin types. Our current research bypasses the need to quantify parameters for each skin type by measuring a “control region” on any subject as well as a “treated region”. This approach can be extended to investigating skin conditions too, and accounts for environmental factors which also affect the skin’s response.

Apart from consistent THz models and measurement protocols, there is also demand for a robust algorithm for parameter extraction, especially as the number of unknown parameters grows. This is not an issue in the single layer structure when it is a simple two-parameter optimization problem. However, when the number of fitting parameters goes beyond 5, classical iteration optimizations may require a precise estimation of the initial values to ensure the convergence. In most cases, the computational complexity would be too large for these algorithms to handle. In this case, heuristic algorithms, optimization methods frequently used for multi-dimensional optimization problems, can be used to balance the accuracy and complexity. For example, the optimization of double Debye parameters has been achieved by using genetic algorithm (GA) by Clegg et al. [69,70] and Ding et al. [71], using a branch and bounding (BB) method by Bao et al. [72,73], and using particle swarm optimization by Yang et al. [74]. A GA was also adopted in the anisotropic SC model fitting by Chen et al. [53]. These algorithms can be efficiently utilized in extracting multiple parameters in a comprehensive skin model.

## 4. Conclusions

The development of skin models is essential to fully understand the interaction between THz waves and skin, which builds the foundation for further investigations of in vivo skin hydration monitoring, drug diffusion monitoring, and medical screening based on skin property changes. In this paper, we have reviewed, summarized and compared the currently used THz models for skin/tissues, including the models for the dielectric properties and the structure of the skin. The structural models first developed from the oversimplified single layer structure to a double-layer model, which better distinguishes the SC and the epidermis. The multilayer model is able to resolve the skin hydration profile in more detail and better displays the hydration changes of skin under different conditions, such as different pressure and occlusion states. The in vivo THz ellipsometer and the anisotropic skin model brings new insights into the biological properties and structure of skin, estimated by the dispersion and birefringence respectively.

As discussed, the use of the model is a trade-off problem between the model accuracy and the result accuracy. Generally, the single layer model is not recommended as it has large errors and provides ambiguous information about the skin, disabling comparison of results from different setups. Choosing between the other three models and the combination with different dielectric models, will be a problem-specific decision that depends on the topic being investigated, the system employed and the sensitivity of the measurement. Theoretically, designing systems capable to provide multiple reflections from uncorrelated geometries are always favorable to enable a more robust and convergent fit. Thus, a more comprehensive model can be solved. For example, the multiple-configuration setup designed for the anisotropic model can also provide a better convergency for the other models. However, at the stage of the current research, most measurements are performed with a unique experimental configuration. The spectrum information is limited to resolve a comprehensive skin structure but a double-layer model or a multiple-layer model can be applied. The former contains fewer unknow parameters and can usually be robustly fitted to explore the properties of SC and epidermis. It can be combined with either the double Debye model or the EMTs to reveal the dielectric properties or the water concentration. On the contrary, the multiple-layer model can only be used with the EMTs by defining a water concentration slope. It is particularly useful when the water depth-profile or the water dynamic changes are of interest. In the circumstances where multiple uncorrelated measurements are available, all the above models can be adopted while the anisotropic model is recommended as the birefringence causes different responses for different polarizations. However, measurements in multiple configurations usually take more time, and the occlusion-induced variation should be more carefully controlled. On the contrary, a non-contact measurement avoids the issue of occlusion, but it is difficult to employ multiple uncorrelated configurations, and is accompanied by other challenges such as misalignment by the curvature of the skin and phase uncertainty, etc. Designing experimental systems with a high depth and water sensitivity can also help robustly and precisely extract the skin parameters from the model fitting, such as employing ATR geometries to enhance the field interacting with the superficial skin. In this case, due to the better field-interaction, the superficial water concentration or gradient in the SC in the double-layer or multiple-layer model can be more sensitively probed. Fundamentally, the improvement of THz systems is essential to overcome these issues and enable a more accurate model to be robustly characterized.

The summarized skin models indicate that THz sensing could be a potential technique to non-invasively reveal complex tissue changes, such as changes in the tissue structure in addition to skin properties and functionality, such as hydration and diffusivity.

## Figures and Tables

**Table 1:** Comparison of different models.

Model	Merits	Limitations
Double Debye model	Physical properties obtained Very little prior knowledge needed	Accuracy for dry tissues is questionable
Effective medium theory	Direct extraction of the water content Very few fitting parameters	Requires a prior knowledge on the biological background EMT assumptions may not be satisfied
Single-layer model	Simple characterization	Oversimplified Cannot distinguish different tissues No comparability between different setups
Double-layer model	Differentiate SC and epidermis Good accuracy	Dielectric models needed
Stratified media model/Fresnel Theory	Clear water concentration distribution Good consistency with Raman spectroscopy	Pre-defined water profile needed More difficult convergence
Anisotropic SC model	Birefringence induced by SC cellular structure considered No dielectric model used Both hydration and structural information obtained	Needs multiple uncorrelated measurements for a convergent fit

## Data Availability

Not applicable.
